# Supplementary material for: Rapid access to discrete and monodisperse block co-oligomers from sugar and terpenoid toward ultrasmall periodic nanostructures
Source: Commun Chem. 2020 Oct 9;3:135. doi: 10.1038/s42004-020-00385-y (PMC9814839; doi:10.1038/s42004-020-00385-y)
Supplement: Supplementary file 2 — Supplementary Information [file 42004_2020_385_MOESM2_ESM.pdf]

S1. Supplementary Methods

S1.1. Materials

Copper(I) bromide (CuBr, >99.995%) was purchased from Sigma Aldrich and used as received. Propargylamine (>95%) and acetic anhydride (>97%) were purchased from Kanto Chemical Co., Inc. and used as received. *N*,*N*,*N'*,*N''*,*N''*-Pentamethyldiethlenetriamine (PMDETA, >98.0%), solanesol (>93.0%), farnesol (mixture of isomers; >95%), DL-*α*-tocopherol (>96%), 1-(3-dimethylaminopropyl)-3-ethylcarbodiimide hydrochloride (EDC·HCl, >98.0%), and 4-dimethylaminopyridine (DMAP, >99.0%) were purchased from Tokyo Chemical Industry Co., Ltd. (TCI) and used as received. Anhydrous tetrahydrofuran (THF, >99.5%; water content, <0.001%), anhydrous DMF (>99.5%; water content, <0.001%), and anhydrous CH_2Cl_2 (>99.5%; water content, <0.001%) were purchased from Kanto Chemical Co., Inc. and used as received. Maltotriose (>97.5%), maltotetraose (>97.0%), and maltohexaose (>97.0%) were purchased from Hayashibara Co., Ltd., and used as received. Dowex® 50WX2 hydrogen form was washed by decantation with MeOH before use. 6-Azidohexanoic acid,[1] propargyl-*β*-D-glucopyranoside (Glc_1-C≡CH),[2] propargyl-*β*-D-maltopyranoside (Glc_2-C≡CH),[3] *N*-maltotriosyl-3-acetamido-1-propyne (Glc_3-C≡CH),[4] and *N*-maltoheptaosyl-3-acetamido-1-propyene (Glc_7-C≡CH)[5] were prepared according to previous reported methods.

S1.2. Instruments

Size exclusion chromatography (SEC). The size exclusion chromatography (SEC) in THF was performed at 40 °C using a Shodex GPC-101 system equipped with a Shodex K-G guard column and a set of two Shodex K-805L columns (linear, 8 mm × 300 mm; bead size, 5 μm; exclusion limit, 4 × 106) at a flow rate of 1.0 mL min^{-1}. The SEC in DMF (containing 0.01 M LiCl) was performed at 40 °C using a Jasco high performance liquid chromatography system (PU-980 Intelligent HPLC pump, CO-965 Column oven, RI-930 Intelligent RI detector, and Shodex DEGAS KT-16) equipped with a Shodex Asahipak GF-310 HQ column (linear, 7.6 mm × 300 mm; pore size, 20 nm; bead size, 5 μm; exclusion limit, 4 × 104) and a Shodex Asahipak GF-7 M HQ column (linear, 7.6 mm × 300 mm; pore size, 20 nm; bead size, 9 μm; exclusion limit, 4 × 107) at a flow rate of 0.6 mL min^{-1}. The number-average molecular weight ($M_{n,SEC}$) and dispersity ($Đ_{SEC}$) of the sample were calculated on the basis of a polystyrene (PSt) calibration.

^{1}H (400 MHz) and ^{13}C NMR (100 MHz). The ^{1}H (400 MHz) and ^{13}C NMR (100 MHz) spectra were recorded using a JEOL JNM-ECS400 instrument.

Matrix-assisted laser desorption ionization time-of-flight mass spectrometry (MALDI-TOF MS). MALDI-TOF MS measurement was performed with the reflector mode using an AB Sciex TOF/TOF 5800 system equipped with a 349-nm Nd:YAG laser. 500 shots were accumulated for the spectra and an acceleration voltage were adjusted between 10 and 30 kV depending on both the molecular weight and the nature of each sample. Samples for the

MALDI-TOF MS were prepared by mixing a THF solution of a sample (5.0 mg mL^{-1}, 1.0 μL) and a matrix (2,5-dihydroxybenzoic acid, 10 mg mL^{-1}, 5.0 μL). For the measurement, the mixed solution of the sample and matrix (1.0 μL) was loaded on a sample plate, which was coated by a solution of NaI (1.0 μL, 1.0 mmol L^{-1}) as the cationic agent in acetone, was used.

Fourier transform infrared spectroscopy (FT-IR). The FT-IR analysis was carried out using a PerkinElmer Frontier MIR spectrometer equipped with a single reflection diamond universal attenuated total reflection (ATR) accessory.

Grazing incidence small-angle X-ray scattering (GISAXS) experiments. GISAXS experiments for thin film samples were performed at BL11 beamline of the ALBA Synchrotron (Barcelona, Spain) or at 3C beamline of the Pohang Accelerator Laboratory (PLA; Pohang, Korea). At the ALBA, the X-ray wavelength and exposure time were 1.00 Å and 30 s, respectively. A PILATUS3 1M (Dectris Ltd., Switzerland) detector, with 981 × 1043 pixels at a pixel size of 172 × 172 μm, and a counter depth of 20 bits (1,048,576 counts), was used for data acquisition. The sample-to-detector distance was calibrated using the scattering patterns of silver behenate. The GISAXS profiles were acquired under ambient condition. At the PLA, scattering data were measured at room temperature in vacuum using X-ray radiation sources with a wavelength of 1.21 Å and a two-dimensional (2D) charge-coupled detector (CCD) (model Rayonix 2D SX 165, Rayonix, Evanston, IL, USA); each scattering image was normally collected for 10 – 30 s. The sample-to-detector distance was calibrated using the scattering patterns of silver behenate.

S1.3 Synthesis

Synthesis of *N*-maltotetraosyl-3-acetamido-1-propyne (Glc_4-C≡CH)

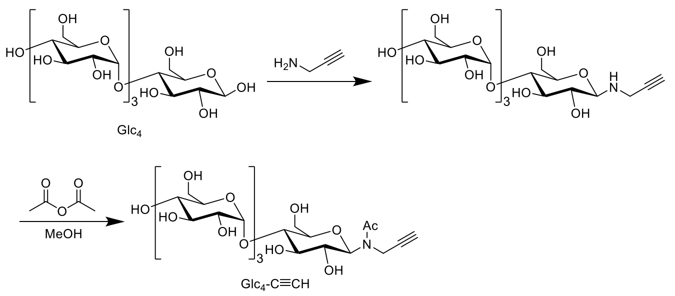

Synthesis of propargyl-functionalized oligosaccharide was conducted as follows (Method A): Maltotetraose (2.00 g, 3.00 mmol) and propargylamine (3.80 mL, 60.0 mmol) were stirred under a nitrogen atmosphere for 72 h. The reaction mixture was dissolved in minimum amount of dry MeOH and precipitated into CH_2Cl_2. The precipitate was filtered and washed with CH_2Cl_2. The obtained precipitate was dissolved in a mixture of acetic anhydride and dry MeOH (1/2(v/v), 100 mL) and stirred for 48 h. The excess of acetic anhydride was removed by co-evaporation with a mixed solvent of toluene and MeOH. The resulting residue was freeze dried to give Glc_4-C≡CH as a white solid (1.86 g, yield: 85.9%).

^{1}H NMR (400 MHz, D_2O): δ (ppm) 5.52 and 5.09 (rotamers, 1H, (HO)C$\underline{H}$N(Ac)), 5.40-5.42 (m, 3H, H-1$^{\underline{GlcII-IV}}$), 3.36-4.30 (m, 26H, $\underline{\text{H-2,-3,-4,-5,-6}^{GlcI-IV}}$, NC$\underline{H_2}$), 2.72 and 2.55 (m, 1H, rotamers, C$\underline{CH}$), 2.20-2.30 (m, 3H, C$\underline{H_3}$),

HRMS (ESI): Calcd.; for $C_{29}H_{47}NO_{21}Na^+$ $[M + Na]^+$ 768.25383, found 768.25360

Synthesis of *N*-maltopentaosyl-3-acetamido-1-propyne (Glc_5-C≡CH)

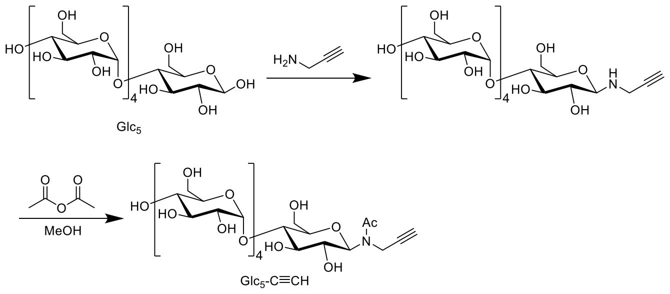

Method A was used for the reaction of maltopehtaose (5.00 g, 6.03mmol) with propargylamine (6.65 g, 121 mmol) and acetic anhydride in MeOH (1/20 (v/v), 100 mL) to give Glc_5-C≡CH as a white solid (5.49 g, yield: 99.0%).

^{1}H NMR (400MHz, D_2O): δ (ppm) 5.54 and 5.09 (rotamers, 1H, C$\underline{H}$N(Ac)), 5.39-5.44 (m, 4H, $\underline{H\text{-}1^{GlcII\text{-}V}}$), 3.36-4.32 (m, 32H, $\underline{H\text{-}2,\text{-}3,\text{-}4,\text{-}5,\text{-}6^{GlcI\text{-}V}}$, NC$\underline{H_2}$), 2.75 and 2.58 (t, 1H, rotamers, *J*= 2.3 Hz, CC$\underline{H}$), 2.32 and 2.24 (s, 3H, C$\underline{H_3}$).

HRMS (ESI): Calcd. For $C_{35}H_{57}NO_{31}Na^+$ $[M + Na]^+$ 930.30779, found 930.30610

Synthesis of *N*-maltohexaosyl-3-acetamido-1-propyne (Glc$_6$-C≡CH)

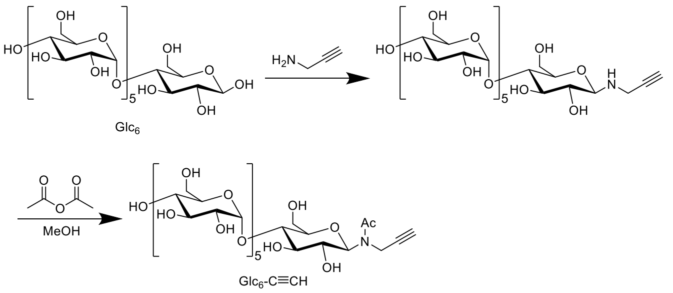

Method A was used for the reaction of maltohexaose (3.00g, 3.03 mmol) with propargylamine (3.88 ml, 60.6 mmol) and acetic anhydride in MeOH (1/20 (v/v), 100 mL) to give Glc$_6$-C≡CH as a white solid (2.80g, yield: 88.4%).

^{1}H NMR (400 MHz, D$_2$O): δ (ppm) 5.54 and 5.09 (rotamers, 1H, C<u>H</u>N(Ac)), 5.39-5.44 (m, 5H, <u>H-1$^{GlcII-VI}$</u>), 3.36-4.32 (m, 38H, <u>H-2,-3,-4,-5,-6$^{GlcI-VI}$</u>, NC<u>H$_2$</u>), 2.75 and 2.58 (t, 1H, rotamers, *J*= 2.3 Hz, CC<u>H</u>), 2.32 and 2.24 (s, 3H, C<u>H$_3$</u>).

HRMS (ESI): Calcd. for $C_{41}H_{67}NO_{31}Na^+$ $[M + Na]^+$ 1092.35947, found 1092.35962.

Synthesis of solanesyl 6-azidohexanoate (N_3-Sol)

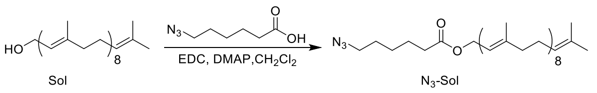

A typical esterification procedure is as follows (method B): 6-Azidohexanoic acid (173 mg, 1.10 mmol) was added to a stirred solution of solanesol (3.00 g, 4.75 mmol), DMAP (871 mg, 7.13 mmol), and EDC·HCl (1.37 g, 7.13 mmol) in anhydrous CH_2Cl_2 (20 mL). After stirring at room temperature for 72 h, the reaction mixture was concentrated and purified by silica gel column chromatography (*n*-hexane/AcOEt = 9/1 (v/v), R_f = 0.44) to give N_3−Sol as a white solid (3.51 g, yield: 95.8%).

^{1}H NMR (400 MHz, $CDCl_3$): δ (ppm) 5.33 (d, 1H, J = 7.2 Hz, $C\underline{H}CH_2O$), 5.12 (t, 8H, J = 6.1 Hz, $C\underline{H}$), 4.60 (d, 2H, J = 6.7 Hz, $C\underline{H_2}O$), 3.27 (t, 2H, J = 7.0 Hz, $C\underline{H_2}N_3$), 2.32 (t, 2H, J = 7.4 Hz, $COC\underline{H_2}$), 1.26-2.10 (m, 68H, $C\underline{H_3}$, $CHC\underline{H_2}C\underline{H_2}$, $CH_2C\underline{H_2}C\underline{H_2}C\underline{H_2}CH_2$).

^{13}C NMR (100 MHz, $CDCl_3$): δ (ppm) 173.6, 142.5, 135.7, 135.2, 135.1, 131.4, 124.5, 124.4, 124.3, 123.7, 118.4, 61.4, 51.4, 39.9, 39.7, 34.2, 29.8, 28.7, 26.9, 26.8, 26.4, 25.9, 24.6, 17.8, 16.6, 16.2.

HRMS (ESI): Calcd. for $C_{51}H_{83}O_2N_3Na^+$ $[M + Na]^+$ 792.63775, found 792.63938.

Synthesis of Glc$_1$-*b*-Sol

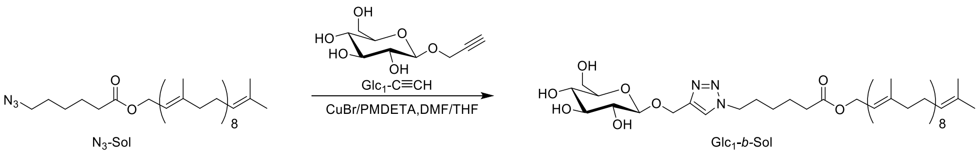

A typical click reaction procedure is as follows (method C). N_3-Sol (1.20 g, 1.56 mmol), Glc$_1$-C≡CH (408 mg, 1.87 mmol), and copper(I) bromide (CuBr; 22.3 mg, 156 μmol) were placed into a Schlenk flask, which was evacuated and back-filled with argon three times. A solution of *N*,*N*,*N'*,*N"*,*N"*-pentamethyldiethylenetriamine (PMDETA;10.3 mg, 59.8 mmol) in a mixed solvent of DMF (5.0 mL) and THF (5.0 mL) was degassed by the argon bubbling, and the mixture was then transferred into the Schlenk flask under an argon atmosphere. The reaction mixture was stirred at 60 °C for 72 h and treated with Dowex® 50WX2 hydrogen form to remove Cu catalyst. The resulting product was purified by reprecipitation from the DMF solution into a mixed solvent of acetonitrile and H_2O (7/3 (v/v)) to give Glc$_1$-*b*-Sol as a white solid (1.03 g, yield: 66.9%). $M_{n,SEC}$ = 2,190; $Đ_{SEC}$ = 1.01 (DMF).

^{1}H NMR (400 MHz, DMF-d_7): See Supplementary Figure 2.

HRMS (ESI): Calcd. for $C_{60}H_{97}O_8N_3Na^+$ $[M+Na]^+$ 1010.71679, found 1010.71725.

Synthesis of maltose-solanesol hybrid polymer (Glc$_2$-*b*-Sol)

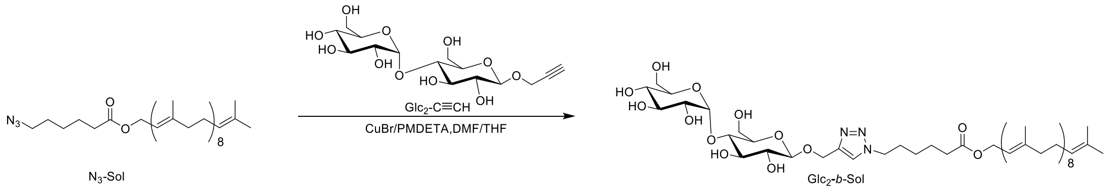

Method C was used for the click reaction of N$_3$-Sol (6.00 g, 7.79 mmol) and Glc$_2$-C≡CH (3.80 g, 9.35 mmol) in a mixed solvent of DMF (25.0 mL) and THF (25.0 mL) with CuBr (112 mg, 779 μmol) and PMDETA (134 mg, 779 μmol) as a catalyst. The resulting product was purified by reprecipitation using THF as a good solvent and a mixed solvent of acetonitrile and H_2O (7/3 (v/v)) as a poor solvent to give Glc$_2$-*b*-Sol as a white solid (6.29 g, yield: 70.2%). $M_{n,SEC}$ = 2,870; $Đ_{SEC}$ = 1.04 (DMF).

^{1}H NMR (400 MHz, DMF-d_7): See Supplementary Figure 3.

HRMS (ESI): Calcd. for $C_{66}H_{107}O_{13}N_3Na^+$ $[M + Na]^+$ 1172.76961, found 1172.76780.

Synthesis of Glc$_3$-*b*-Sol

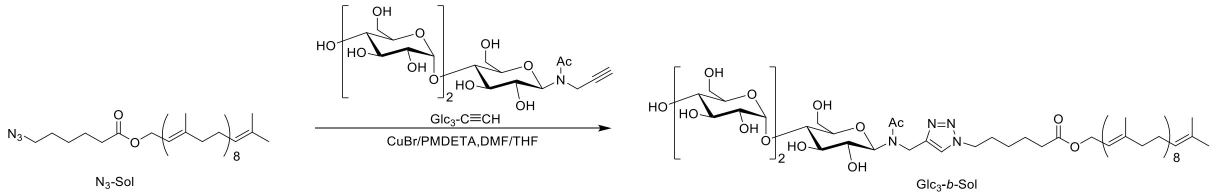

Method C was used for the click reaction of N$_3$-Sol (1.00 g, 1.30 mmol) and Glc$_3$-C≡CH (908 mg, 1.56 mmol) in a mixed solvent of DMF (10.0 mL) and THF (6.0 mL) with CuBr (18.6 mg, 130 μmol) and PMDETA (22.5 mg, 130 μmol) as a catalyst. The resulting product was purified by reprecipitation using THF as a good solvent and a mixed solvent of acetonitrile and H_2O (7/3 (v/v)) as a poor solvent to give Glc$_3$-*b*-Sol as a white solid (1.67 g, yield: 95.1%). $M_{n,SEC}$ = 3,470; $Đ_{SEC}$ = 1.02 (DMF).

^{1}H NMR (400 MHz, DMF-d_7): Supplementary See Figure 4.

HRMS (ESI): Calcd. for $C_{74}H_{120}O_{18}N_4Na^+$ $[M + Na]^+$ 1375.84898, found 1375.85015.

Synthesis of Glc$_4$-*b*-Sol

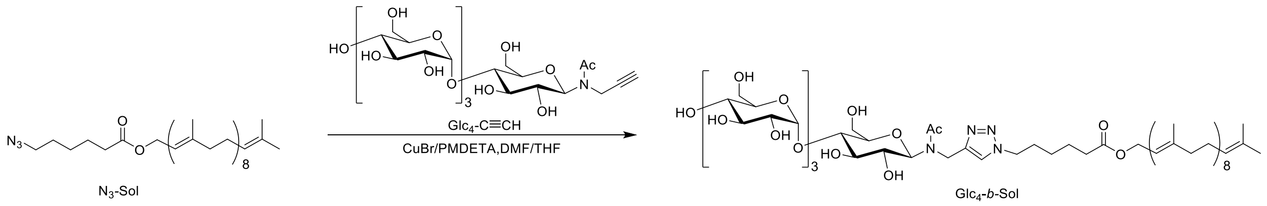

Method C was used for the click reaction of N_3-Sol (500 mg, 0.650 mmol) and Glc$_4$-C≡CH (581 mg, 0.780 mmol) in a mixed solvent of DMF (10.0 mL) and THF (6.0 mL) with CuBr (9.3 mg, 65 μmol) and PMDETA (11.3 mg, 65 μmol) as a catalyst. The resulting product was purified by reprecipitation using THF as a good solvent and a mixed solvent of acetonitrile and H_2O (7/3 (v/v)) as a poor solvent to give Glc$_4$-*b*-Sol as a white solid (0.873 g, yield:88.3%). $M_{n,SEC}$ = 3,470; $Đ_{SEC}$ = 1.03 (DMF).

^{1}H NMR (400 MHz, DMF-d_7): See Supplementary Figure 5.

HRMS (ESI): Calcd. for $C_{80}H_{131}O_{23}N_4^+$ $[M + H]^+$ 1515.91986, found 1515.92241.

Synthesis of Glc$_5$-*b*-Sol

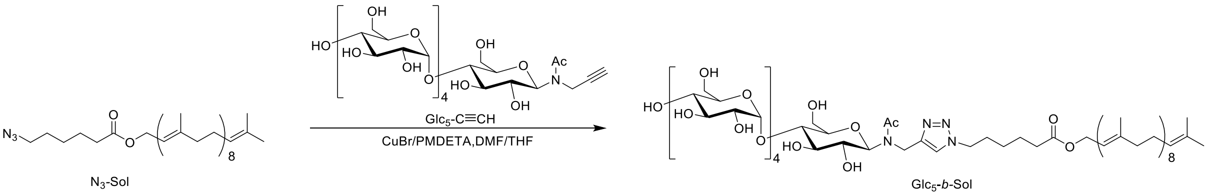

Method C was used for the click reaction of N$_3$-Sol (150 mg, 0.194 mmol) and Glc$_5$-C≡CH (212 mg, 0.234 mmol) in a mixed solvent of DMF (1.5 mL) and THF (1.5 mL) with CuBr (2.8 mg, 20 μmol) and PMDETA (3.4 mg, 20 μmol) as a catalyst. The resulting product was purified by reprecipitation using DMF as a good solvent and a mixed solvent of acetonitrile and H_2O (7/3 (v/v)) as a poor solvent to give Glc$_5$-*b*-Sol as a white solid (176 mg, yield: 54.0%). $M_{n,SEC}$ = 3734; $Đ_{SEC}$ = 1.02 (DMF).

^{1}H NMR (400 MHz, DMF-d_7): See Supplementary Figure 6.

HRMS (ESI): Calcd. for $C_{86}H_{140}O_{28}N_4Na^+$ $[M + Na]^+$ 1699.95693, found 1699.95463.

Synthesis of Glc_6-*b*-Sol

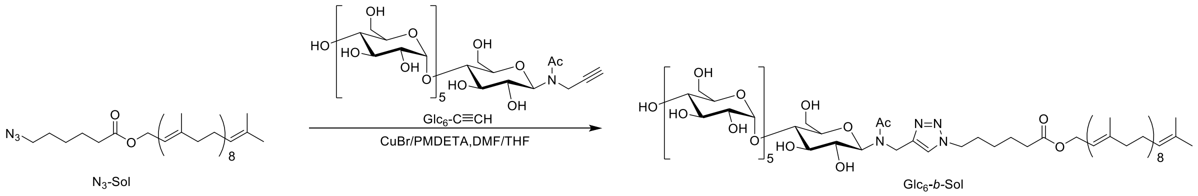

Method C was used for the click reaction of N_3-Sol (150 mg, 0.194 mmol) and Glc_6-C≡CH (173 mg, 0.162 mmol) in a mixed solvent of DMF (1.5 mL) and THF (1.5 mL) with CuBr (2.3 mg, 16.2 μmol) and PMDETA (2.8 mg, 16.2 μmol) as a catalyst. The resulting product was purified by reprecipitation using DMF as a good solvent and acetone as a poor solvent to give Glc_6-*b*-Sol as a white solid (212 mg, yield: 59.3%). $M_{n,SEC}$ = 4,140; $Đ_{SEC}$ = 1.04 (DMF).

^{1}H NMR (400 MHz, DMF-d_7): See Supplementary Figure 7.

HRMS (ESI): Calcd. for $C_{92}H_{151}O_{33}N_4^+$ $[M + H]^+$ 1840.02551, found 1840.03129.

Synthesis of Glc$_7$-*b*-Sol

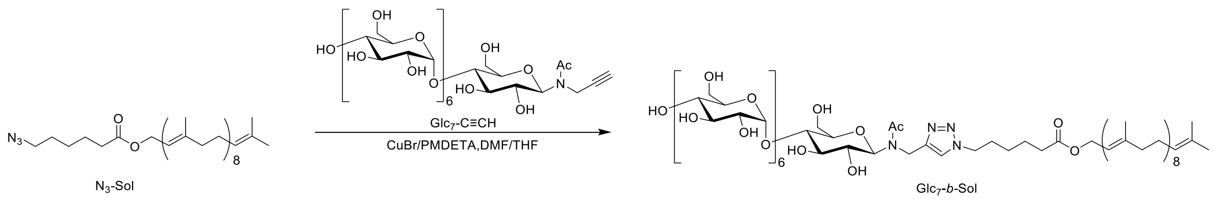

Method C was used for the click reaction of N_3-Sol (150 mg, 0.194 mmol) and Glc$_7$-C≡CH (288 mg, 0.234 mmol) in a mixed solvent of DMF (1.5 mL) and THF (1.5 mL) with CuBr (2.8 mg, 19.5 μmol) and PMDETA (3.4 mg, 19.5 μmol) as a catalyst. The resulting product was purified by reprecipitation using DMF as a good solvent and a mixed solvent of acetonitrile and H_2O (7/3 (v/v)) as a poor solvent. Further purification by reprecipitation using DMF as a good solvent and acetone as a poor solvent gave Glc$_7$-*b*-Sol as a white solid (251 mg, yield: 64.3%). $M_{n,SEC}$ = 5,150; $Đ_{SEC}$ = 1.04 (DMF).

^{1}H NMR (400 MHz, DMF-d_7): See Supplementary Figure 8.

HRMS (ESI): Calcd. for $C_{98}H_{160}O_{38}N_4Na_2^{2+}$ [M + 2Na]$^{2+}$ 1023.52475, found 1023.52689.

Synthesis of N_3-Toc

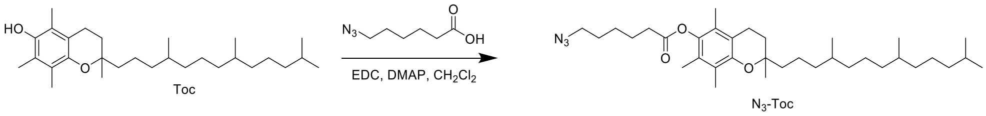

Method B was used for the reaction of DL-α-tocopherol (7.00 g, 16.26 mmol) and 6-azidohexanoic acid (3.32 g, 21.12 mmol) with DMAP (2.58 g, 21.12 mmol) and EDC·HCl (4.05 g, 21.12 mmol) in anhydrous CH_2Cl_2 (50 mL). The crude was purified by silica gel column chromatography (*n*-hexane/AcOEt = 1/4 (v/v), R_f = 0.13) to give N_3-Toc as a pale-yellow viscous liquid (1.90 g, yield: 57.7%).

^{1}H NMR (400 MHz, $CDCl_3$): δ (ppm) 3.31 (t, 2H, J = 7.2 Hz, $N_3C\underline{H_2}$), 2.68-2.52 (m, 4H, $COC\underline{H_2}$, Ar–$C\underline{H_2}$), 2.12-1.92 (m, 9H, Ar–$C\underline{H_3}$), 1.90-0.96 (m, 37H, $\underline{H^{Toc}}$, $N_3CH_2C\underline{H_2}C\underline{H_2}C\underline{H_2}$), 0.94-0.78 (m, 12H, $C\underline{H_3}$).

^{13}C NMR (100 MHz, $CDCl_3$): δ (ppm) 172.2, 149.5, 140.5, 126.7, 125.0, 123.2, 117.5, 75.1, 51.3, 39.5, 39.4, 37.6, 37.5, 37.5, 37.4, 34.0, 32.9, 32.8, 28.7, 28.1, 26.5, 25.0, 24.8, 24.6, 22.8, 22.8, 21.1, 20.7, 19.9, 19.8, 19.7, 19.7, 13.1, 12.27, 11.96.

HRMS (ESI): Calcd. for $C_{35}H_{59}O_3N_3Na^+$ $[M + Na]^+$ 592.44486, found 592.44528.

Synthesis of Glc_1-*b*-Toc

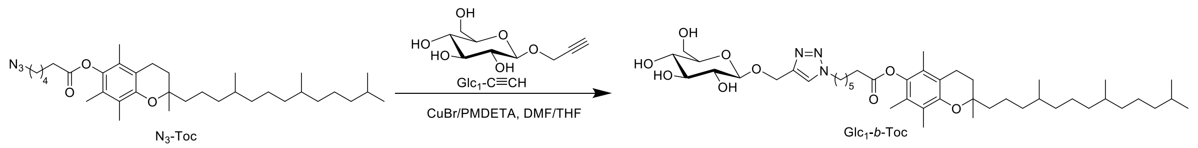

Method C was used for the click reaction of N_3-Toc (2.00 g, 3.51 mmol) and Glc_1-C≡CH (512 mg, 2.35 mmol) in a mixed solvent of DMF (10 mL) and THF (10 mL) with CuBr (101 mg, 0.71 mmol) and PMDETA (122 mg, 0.71 mmol) as a catalyst. The resulting product was purified by silica gel column chromatography (CH_2Cl_2/MeOH = 9/1 (v/v), R_f = 0.27) to give Glc_1-*b*-Toc as a white solid (1.48 g, yield: 53.5%). $M_{n,SEC}$ = 1790; $Đ_{SEC}$ = 1.01 (DMF).

^{1}H NMR (400 MHz, DMSO-*d*$_6$): See Supplementary Figure 11.

HRMS (ESI): Calcd. for $C_{44}H_{73}O_9N_3Na^+$ $[M + Na]^+$ 810.52390, found 810.52444.

Synthesis of Glc$_2$-*b*-Toc

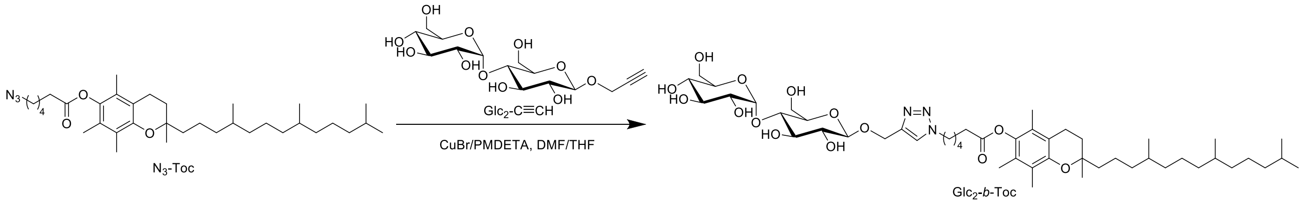

Method C was used for the click reaction of N$_3$-Toc (800 mg, 1.40 mmol) and Glc$_2$-C≡CH (357 mg, 0.94 mmol) in a mixed solvent of DMF (5 mL) and THF (5 mL) with CuBr (44 mg, 0.28 mmol) and PMDETA (49 mg, 0.28 mmol) as a catalyst. The resulting product was purified by silica gel column chromatography (CH_2Cl_2/MeOH = 4/1 (v/v), R_f = 0.35) to give Glc$_2$-*b*-Toc as a yellow solid (320 mg, yield: 31.3%). $M_{n,SEC}$ = 2240; $Đ_{SEC}$ = 1.02 (DMF).

^{1}H NMR (400 MHz, DMSO-d_6): See Supplementary Figure 12.

HRMS (ESI): Calcd. for $C_{58}H_{97}O_{19}N_3H^+$ $[M + H]^+$ 1153.677415, found 1153.67595.

Synthesis of N_3-Far

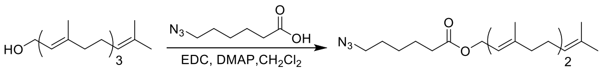

Far N3-Far

Method B was used for the reaction of farnesol (mixture of isomers; 6.00 g, 27.0 mmol) and 6-azidohexanoic acid (5.52 g, 35.1 mmol) with DMAP (4.29 g, 35.1 mmol) and EDC·HCl (6.74 g, 35.1 mmol) in anhydrous CH_2Cl_2 (50 mL). The crude was purified by silica gel column chromatography (*n*-hexane/AcOEt = 9/1 (v/v), R_f = 0.31) to give N_3-Far as a pale-yellow viscous liquid (8.21 g, yield: 84.2%).

^{1}H NMR (400 MHz, $CDCl_3$): δ (ppm) 5.35 (t, 1H, J = 7.2 Hz, $C\underline{H}CH_2O$), 5.20-5.04 (m, 2H, $C\underline{H}$), 4.70-4.52 (m, 2H, $C\underline{H_2}O$), 3.28 (t, 2H, J = 7.2 Hz, $C\underline{H_2}N_3$), 2.42-2.26 (m, 2H, $COC\underline{H_2}$), 2.22-1.92 (m, 8H, $CHC\underline{H_2}C\underline{H_2}$), 1.83-1.51 (m, 16H, $C\underline{H_3}$, $CH_2C\underline{H_2}CH_2C\underline{H_2}CH_2$), 1.49-1.31 (m, 2H, $CH_2CH_2C\underline{H_2}CH_2CH_2$).

^{13}C NMR (100 MHz, $CDCl_3$): δ (ppm) 173.6 ($\underline{C}O$), 142.5 ($OCH_2CH\underline{C}$), 135.6 ($OCH_2CHCCH_2CH_2CH\underline{C}$), 131.5 ($\underline{C}(CH_3)_2$), 124.3 and 123.8 ($OCH_2CHCCH_2CH_2\underline{C}HCCH_2CH_2\underline{C}HC$), 118.3 ($OCH_2\underline{C}HC$), 61.4 ($OCH_2\underline{C}HC$), 51.3 ($N_3\underline{C}H_2$), 39.8 and 39.6 ($C(CH_3)\underline{C}H_2$), 34.2 ($CO\underline{C}H_2$), 28.7 ($N_3CH_2\underline{C}H_2$), 26.8 ($N_3CH_2CH_2\underline{C}H_2$), 26.3 and 26.3 ($CH_2\underline{C}H_2CH$), 24.6 ($COCH_2\underline{C}H_2$), 24.5 ($C(\underline{C_{trans}}H_3)(C_{cis}H_3)$), 17.8 ($C(C_{trans}H_3)(\underline{C_{cis}}H_3)$), 16.6 and 16.1 ($C(\underline{C}H_3)CH_2$).

HRMS (ESI): Calcd. for $C_{21}H_{35}O_2N_3Na^+$ $[M + Na]^+$ 384.26215, found 384.26257.

Synthesis of Glc_1-*b*-Far

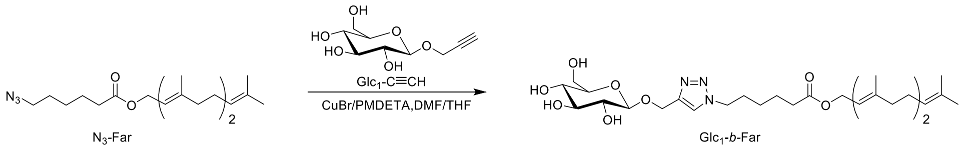

Method C was used for the click reaction of N_3-Far (420 mg, 1.16 mmol) and Glc_1-C≡CH (350 mg, 1.61 mmol) in a mixed solvent of DMF (5.0 mL) and THF (5.0 mL) with CuBr (20.0 mg, 139 μmol) and PMDETA (122 mg, 111 μmol) as a catalyst. The resulting product was purified by reprecipitation using DMF as a good solvent and water as a poor solvent to give to give Glc_1-*b*-Far as a white solid (280 mg, yield: 42.0%). $M_{n,SEC}$ = 1543; $Đ_{SEC}$ = 1.015 (DMF).

^{1}H NMR (400 MHz, DMSO-d_6): See Supplementary Figure 14.

HRMS (ESI): Calcd. for $C_{30}H_{50}O_8N_3Na^+$ $[M + Na]^+$ 580.35924, found 580.36002.

S2. Supplementary Data

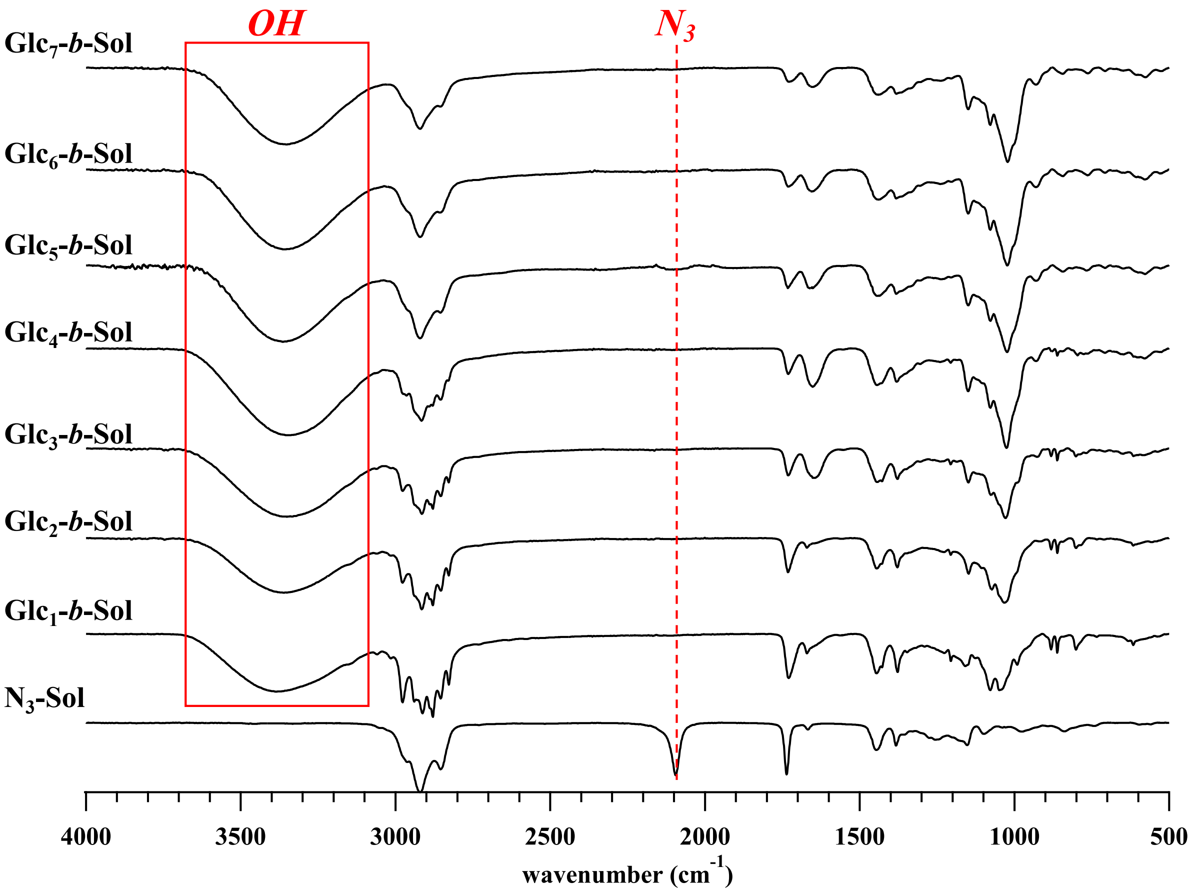

Supplementary Figure 1. FT-IR spectra of N_3-Sol and Glc$_n$-*b*-Sol (n = 1 – 7).

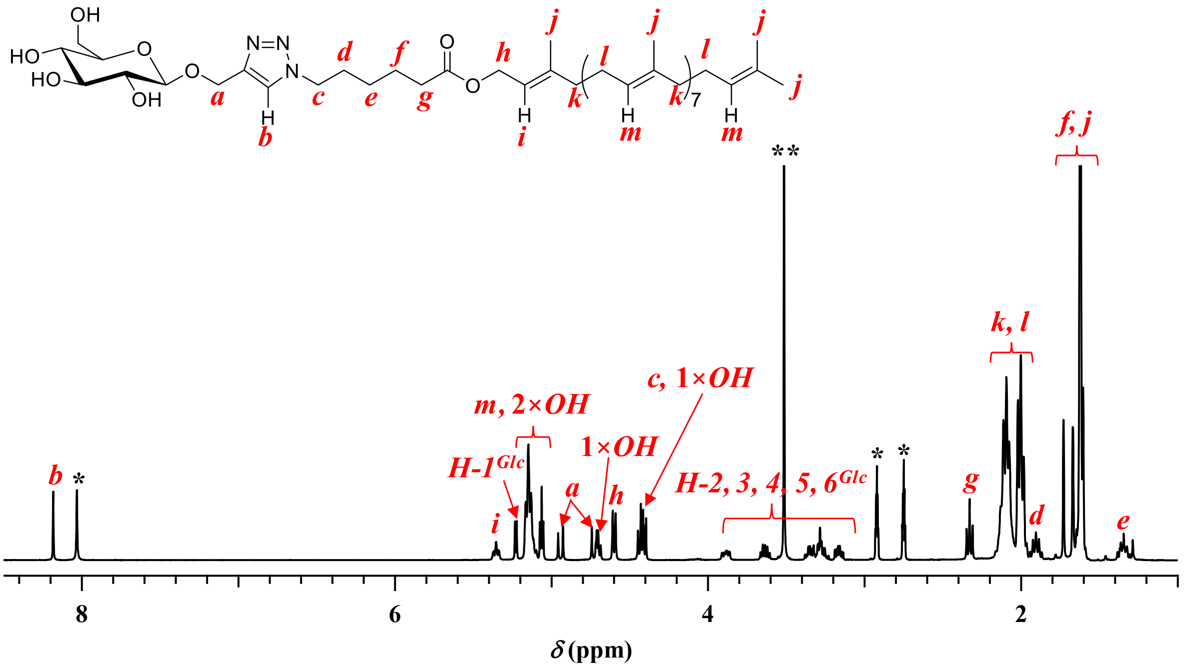

Supplementary Figure 2. ^{1}H NMR spectrum of Glc1-*b*-Sol in DMF-d_7.

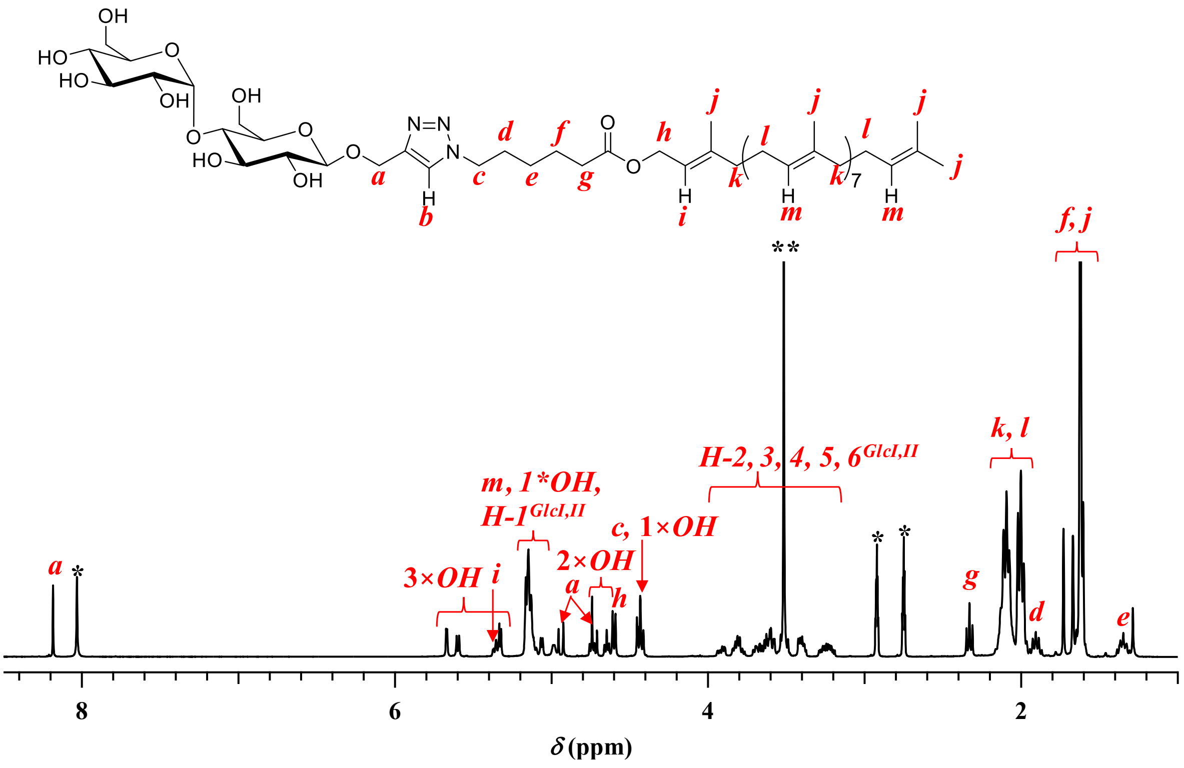

Supplementary Figure 3. ^{1}H NMR spectrum of Glc_2-*b*-Sol in DMF-d_7.

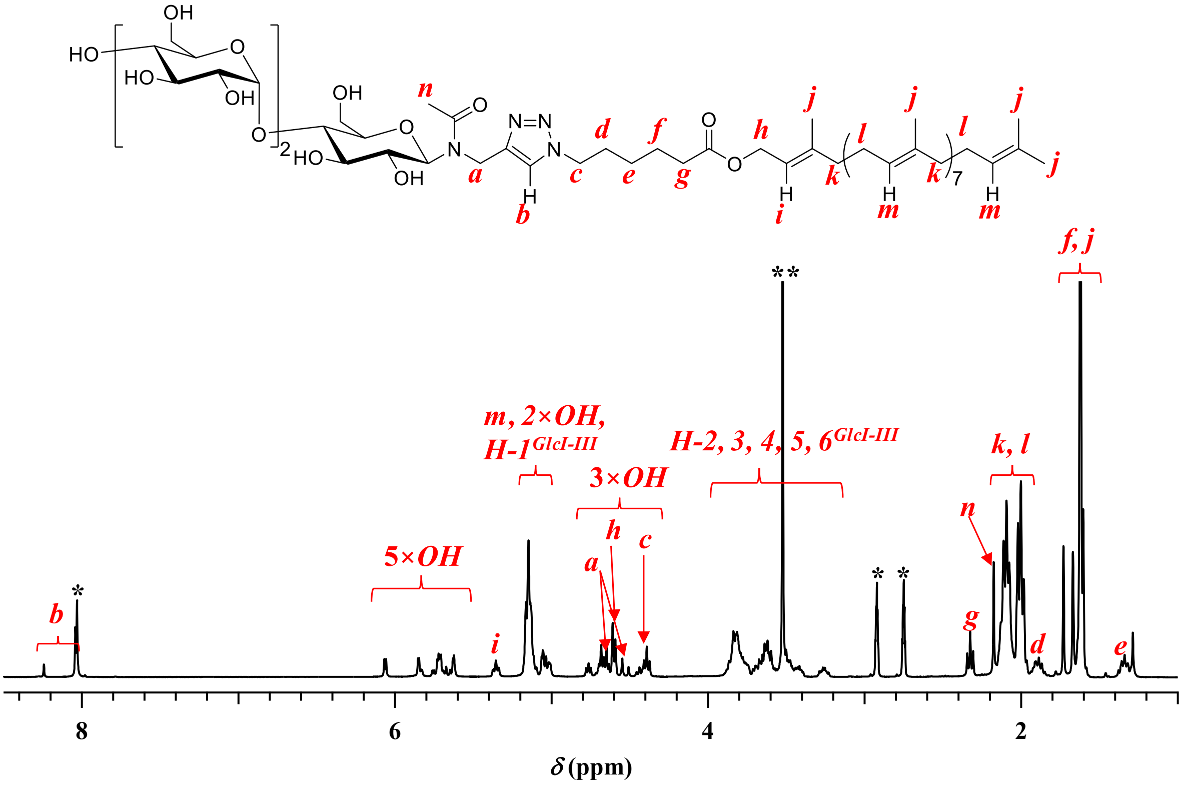

Supplementary Figure 4. ^{1}H NMR spectrum of Glc_3-*b*-Sol in DMF-d_7.

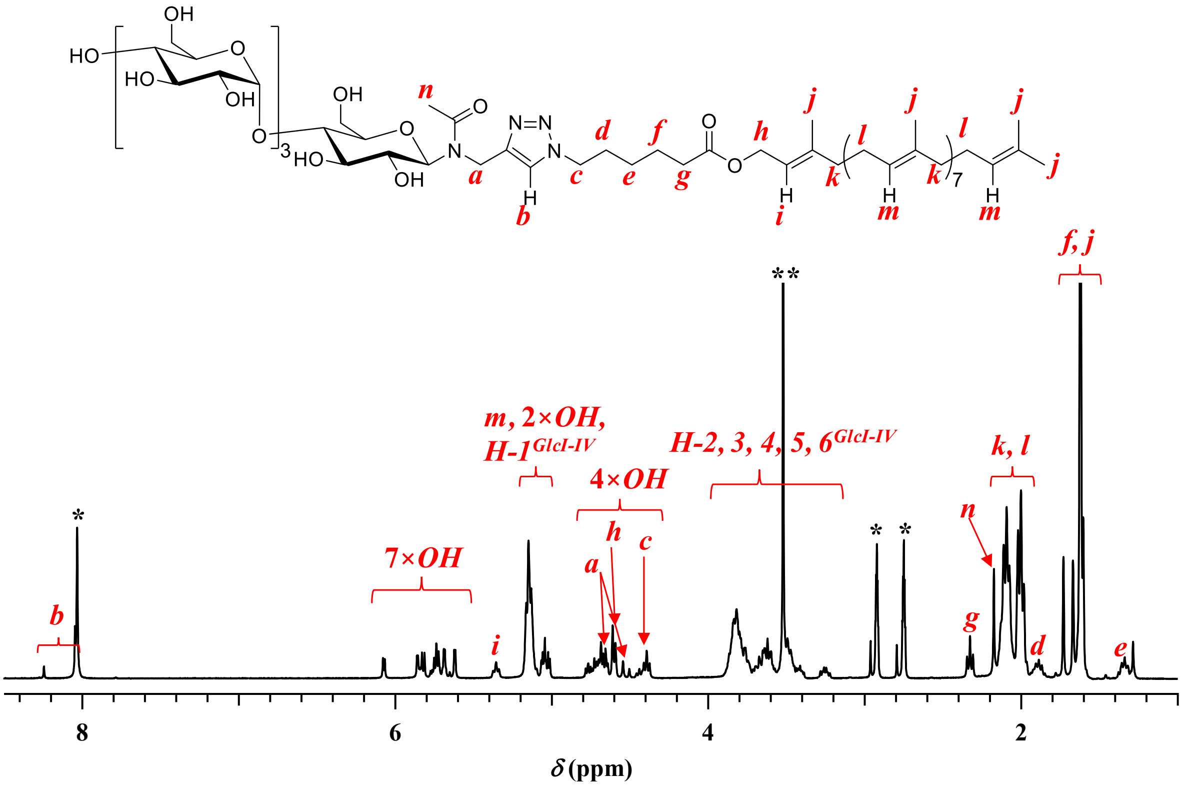

Supplementary Figure 5. ^{1}H NMR spectrum of Glc4-*b*-Sol in DMF-d_7.

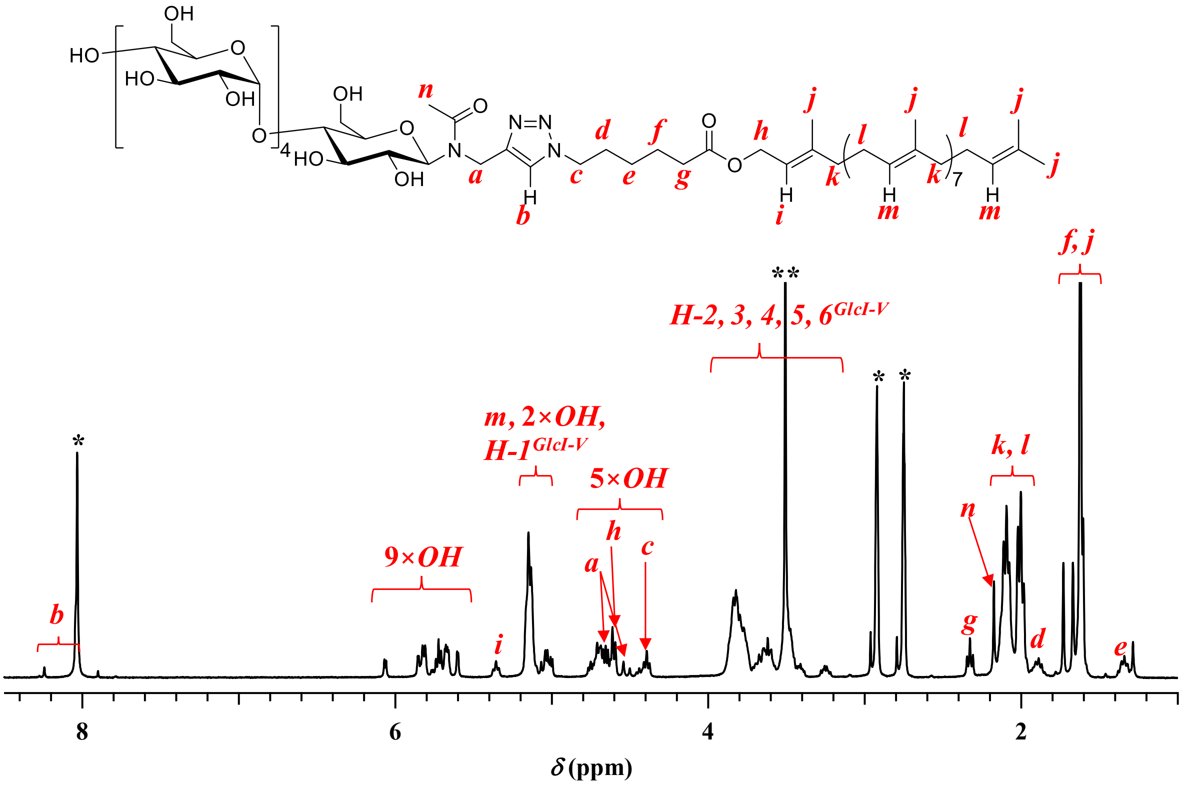

Supplementary Figure 6. ^{1}H NMR spectrum of Glc5-*b*-Sol in DMF-d_7.

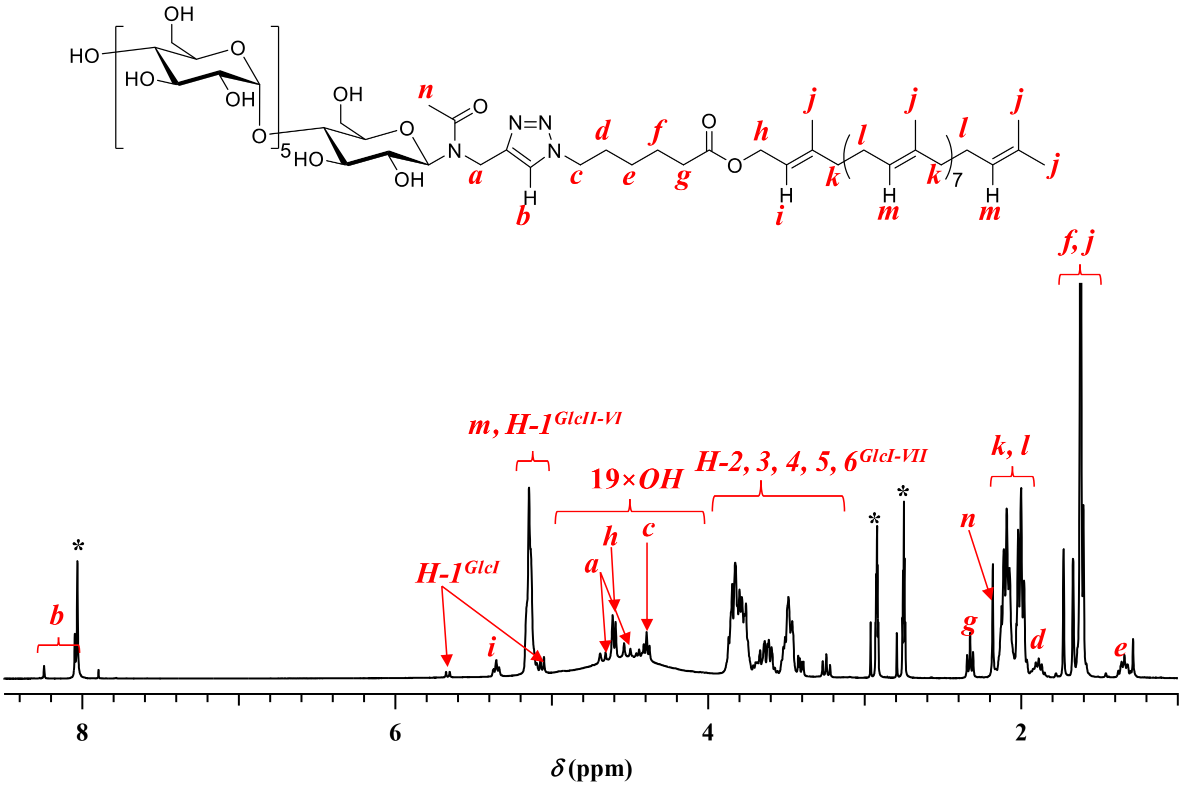

Supplementary Figure 7. ^{1}H NMR spectrum of Glc_6-*b*-Sol in DMF-d_7.

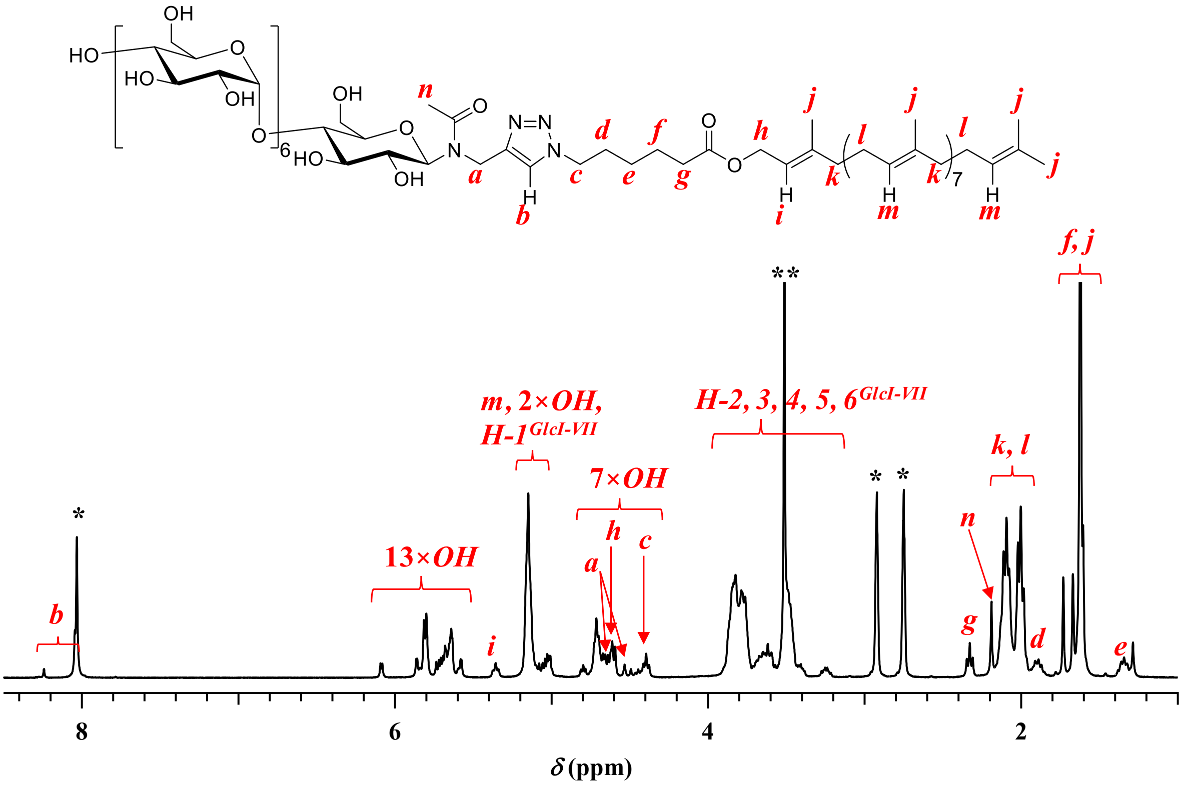

Supplementary Figure 8. ^{1}H NMR spectrum of Glc_1-*b*-Sol in DMF-d_7.

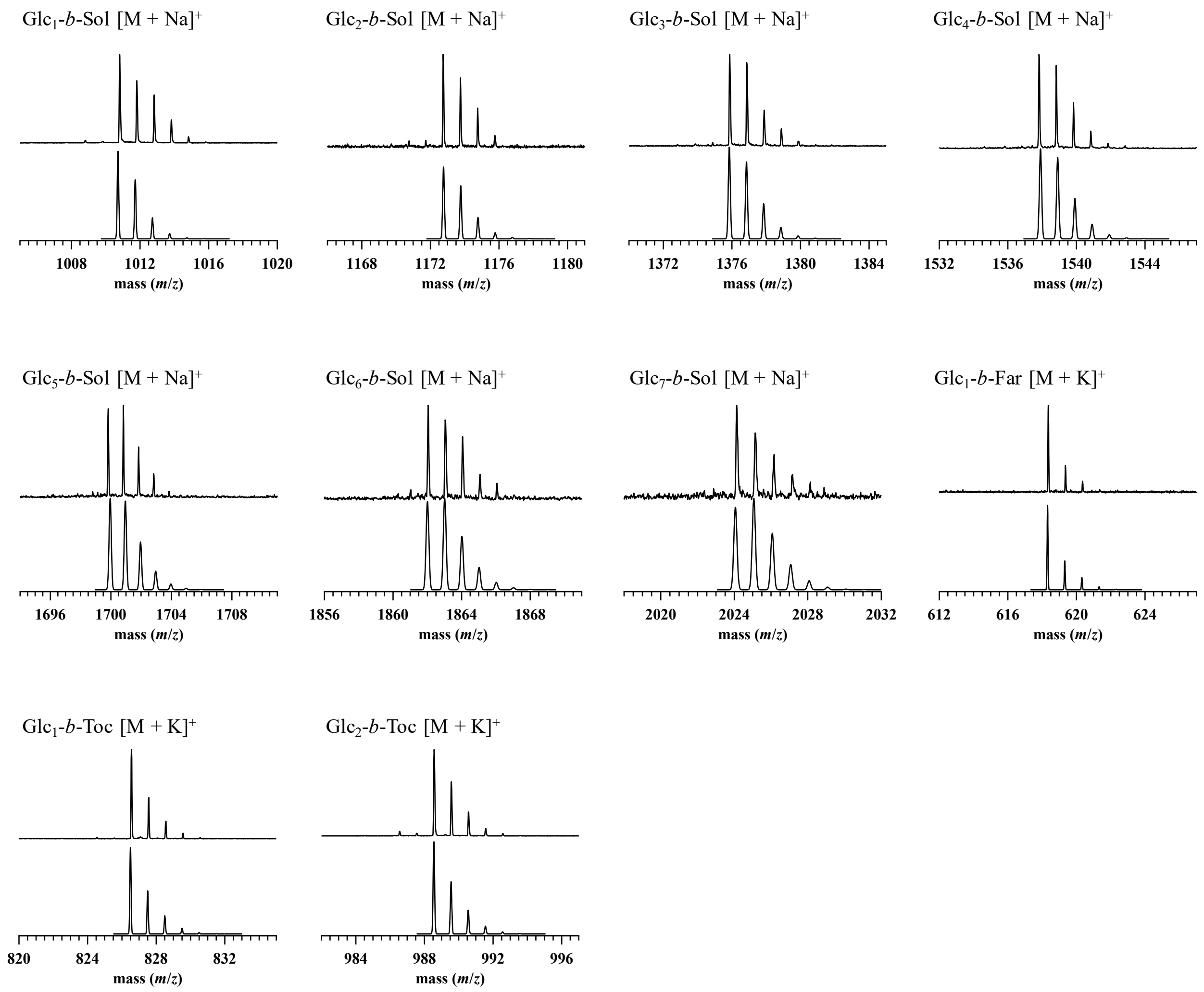

Supplementary Figure 9. MALDI-TOF mass spectral analysis. Isotope distributions of the MALDI-TOF mass spectra of Glc*n*-*b*-Sol (*n* = 1 – 7; sodium adduct), Glc1-*b*-Far (potassium adduct), and Glc*n*-*b*-Toc (*n* = 1 and 2; potassium adduct). The upper and lower represent the experimental and simulated spectra, respectively.

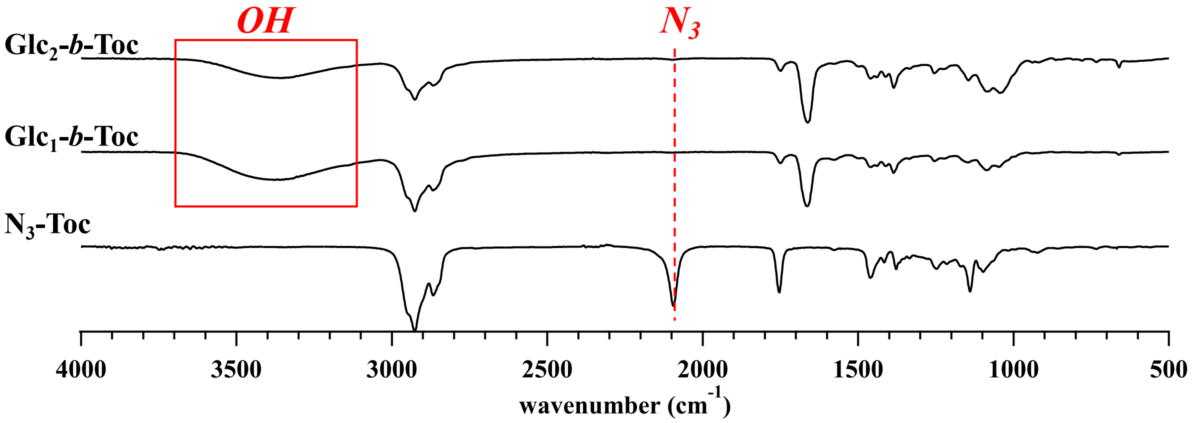

Supplementary Figure 10. FT-IR spectra of N_3-Toc and Glc$_n$-*b*-Toc (n = 1 and 2).

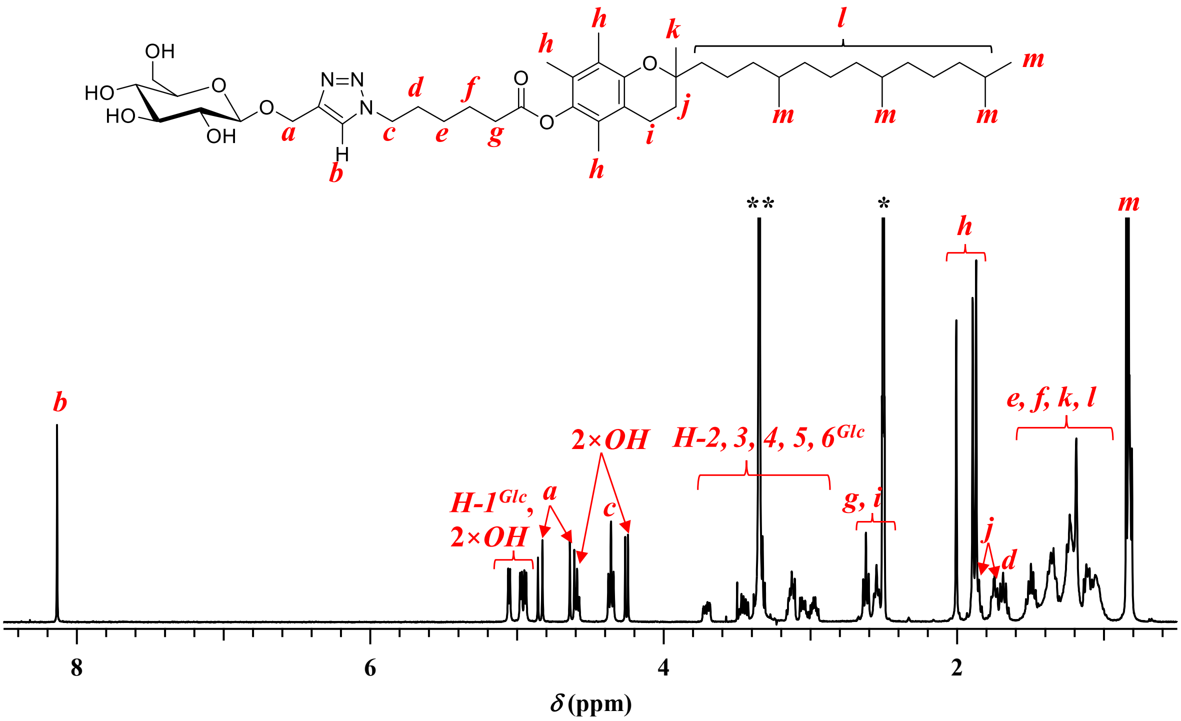

Supplementary Figure 11. ^{1}H NMR spectrum of Glc$_1$-*b*-Toc in DMSO-d_6.

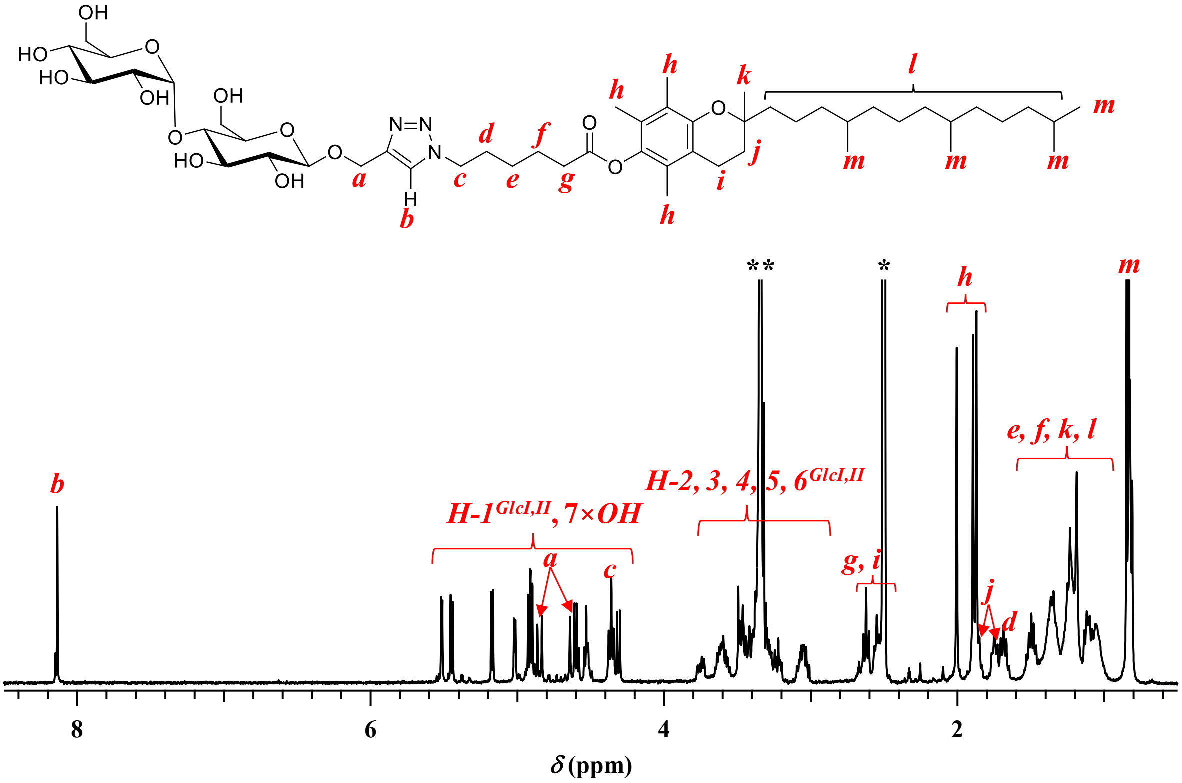

Supplementary Figure 12. ^{1}H NMR spectrum of Glc$_2$-*b*-Toc in DMSO-d_6.

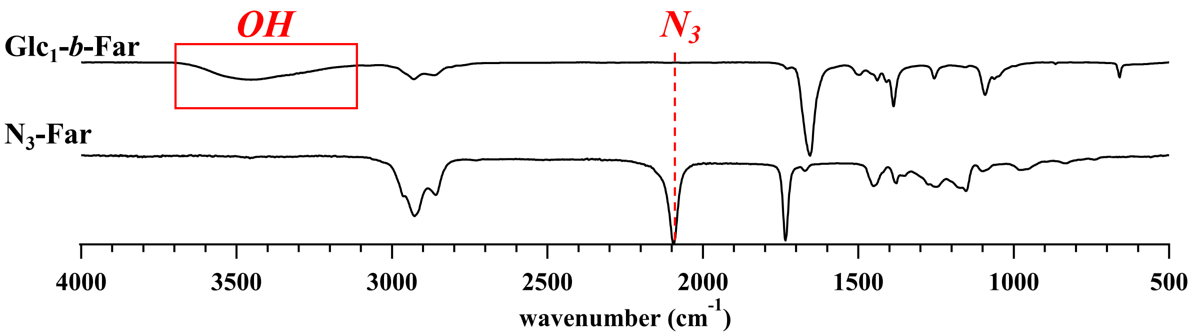

Supplementary Figure 13. FT-IR spectra of N_3-Far and Glc_1-*b*-Far.

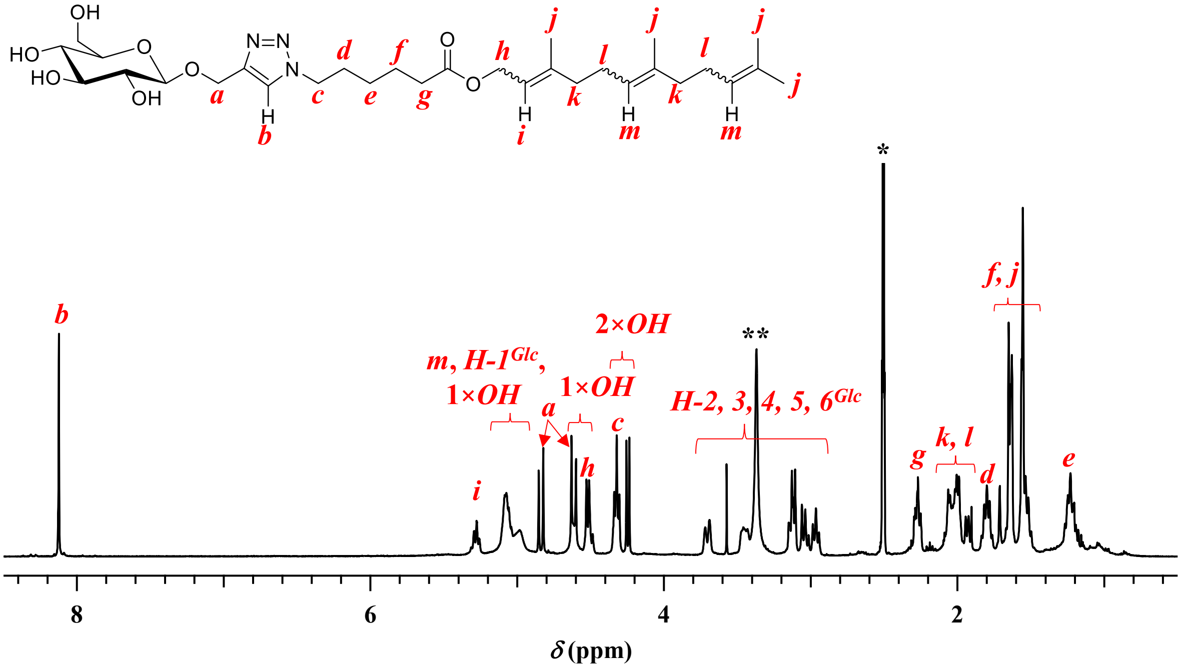

Supplementary Figure 14. ^{1}H NMR spectrum of Glc_1-*b*-Far in DMSO-d_6.

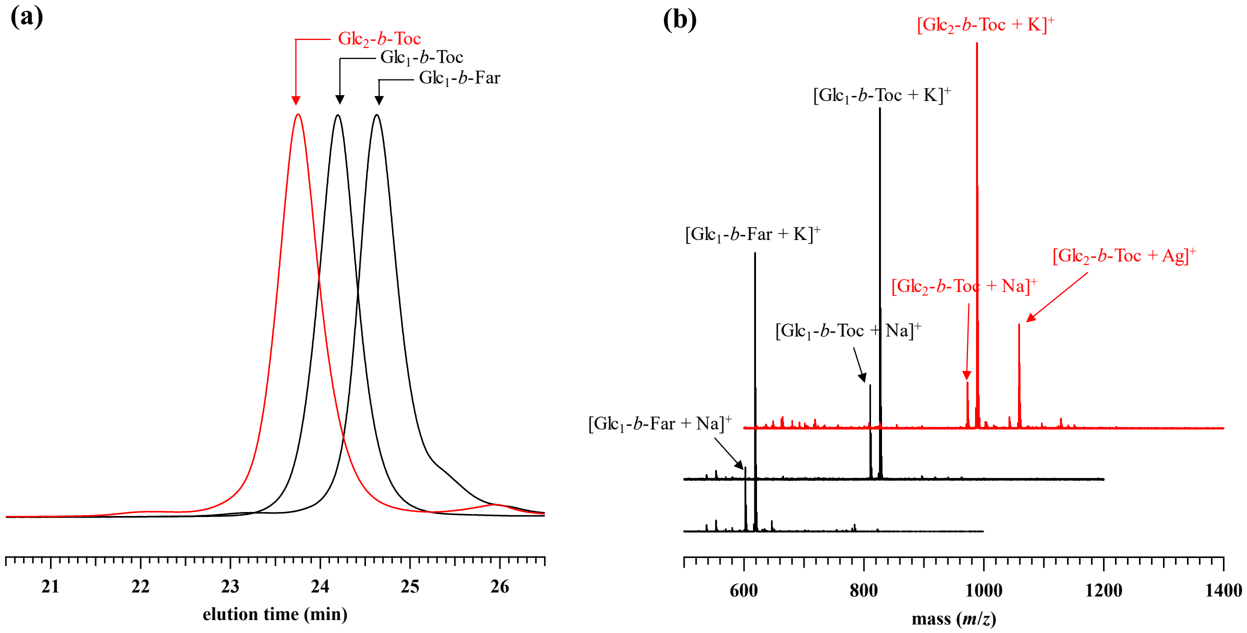

Supplementary Figure 15. (a) SEC traces (eluent, DMF containing 0.01 M LiCl) and (b) MALDI-TOF mass spectra of Glc_1-*b*-Far and Glc_n-*b*-Toc (n = 1 and 2).

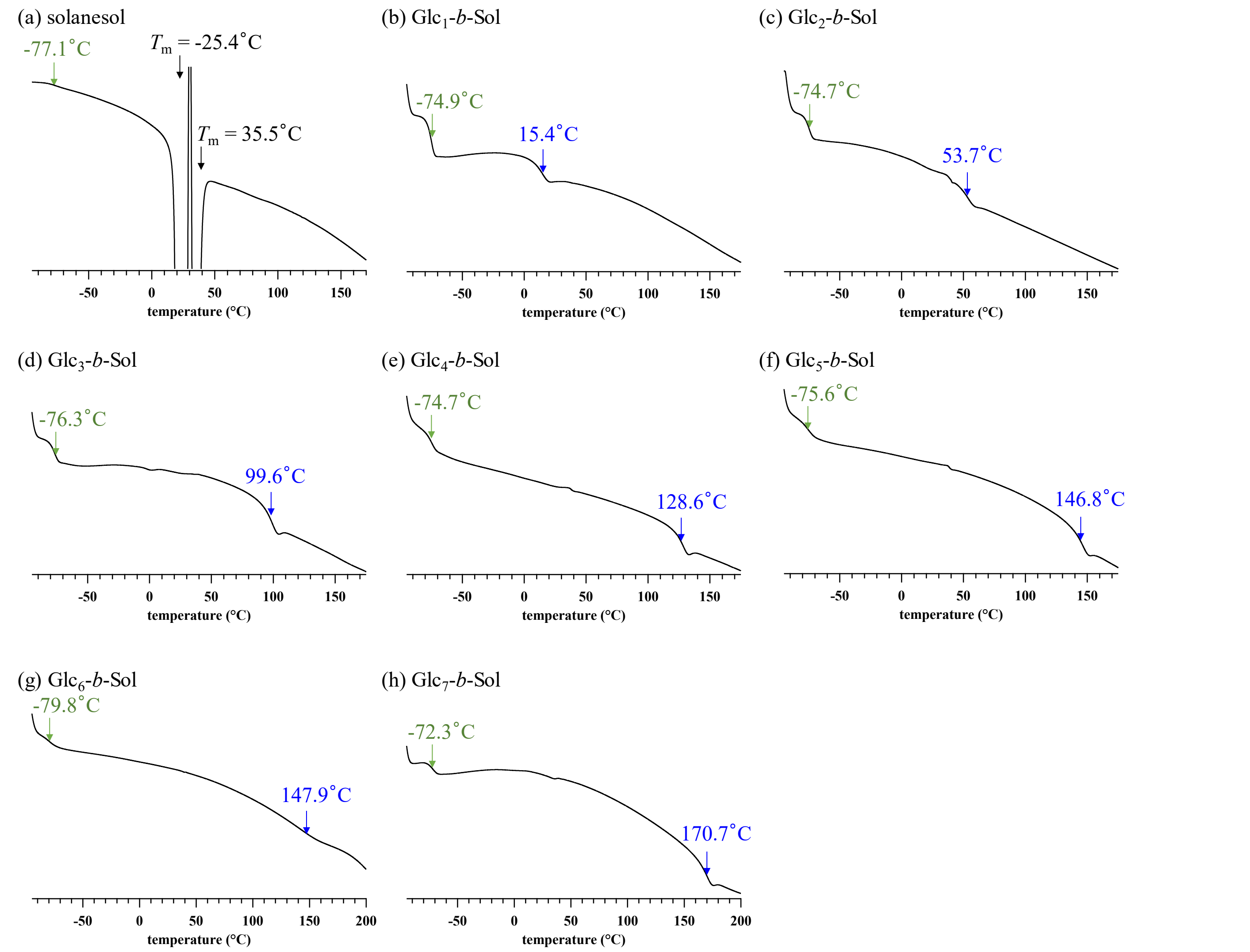

Supplementary Figure 16. DSC analysis of Glc_n-*b*-Sol. DSC traces of solanesol and Glc_n-*b*-Sol (n = 1 – 7) during the second heating run (heating and cooling rate, 10 °C min^{-1}). The temperature value with green and blue colors represent the T_g for the solanesol and sugar segments, respectively.

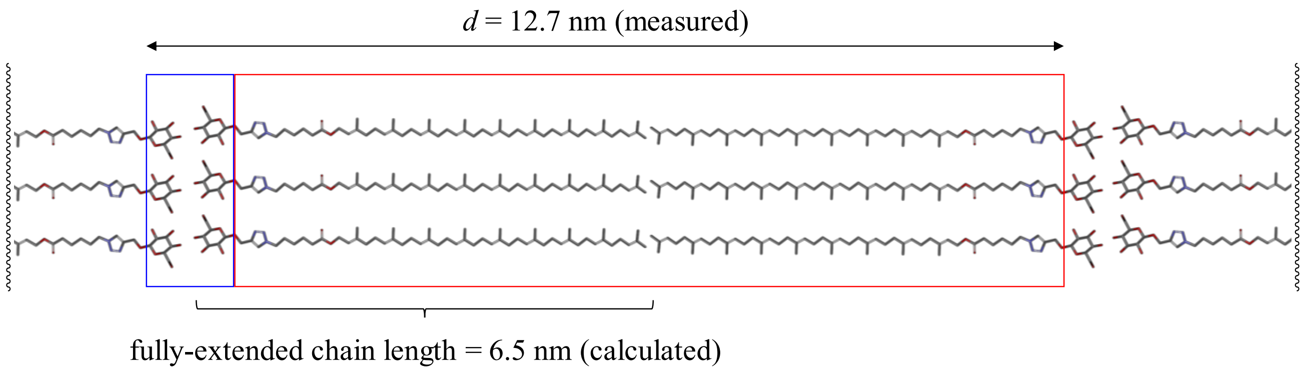

Supplementary Figure 17. Schematic illustration for a possible molecular packing of Glc_1-*b*-Sol molecules in the lamellar morphology. Since the fully-extended chain length of Glc_1-*b*-Sol is ca. 6.5

nm, Glc$_1$-*b*-Sol molecules seem to be crystallized as a dimer in a head-to-head configuration.

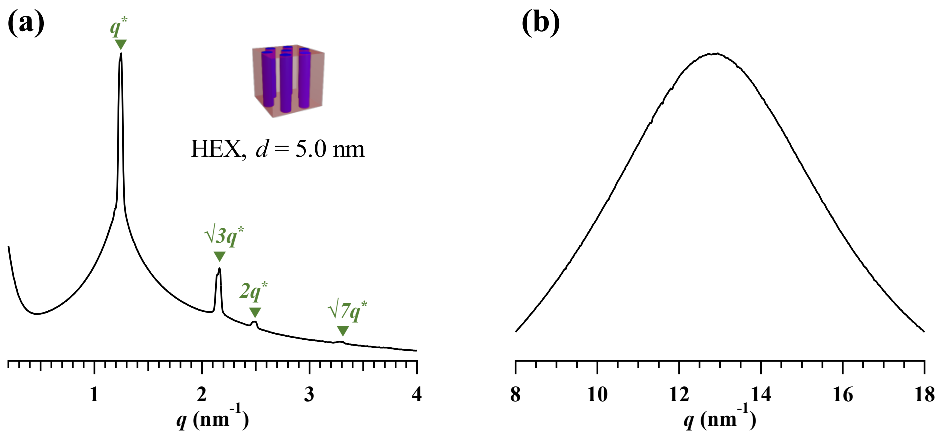

Supplementary Figure 18. *In-situ* X-ray analysis of Glc$_1$-*b*-Sol. (a) *In-situ* SAXS and (b) WAXS profiles of Glc$_1$-*b*-Sol measured at 180 °C.

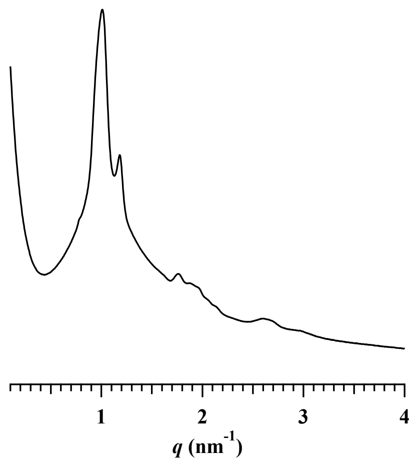

Supplementary Figure 19. SAXS profile of Glc$_2$-*b*-Sol annealed after 130 °C for 36 h.

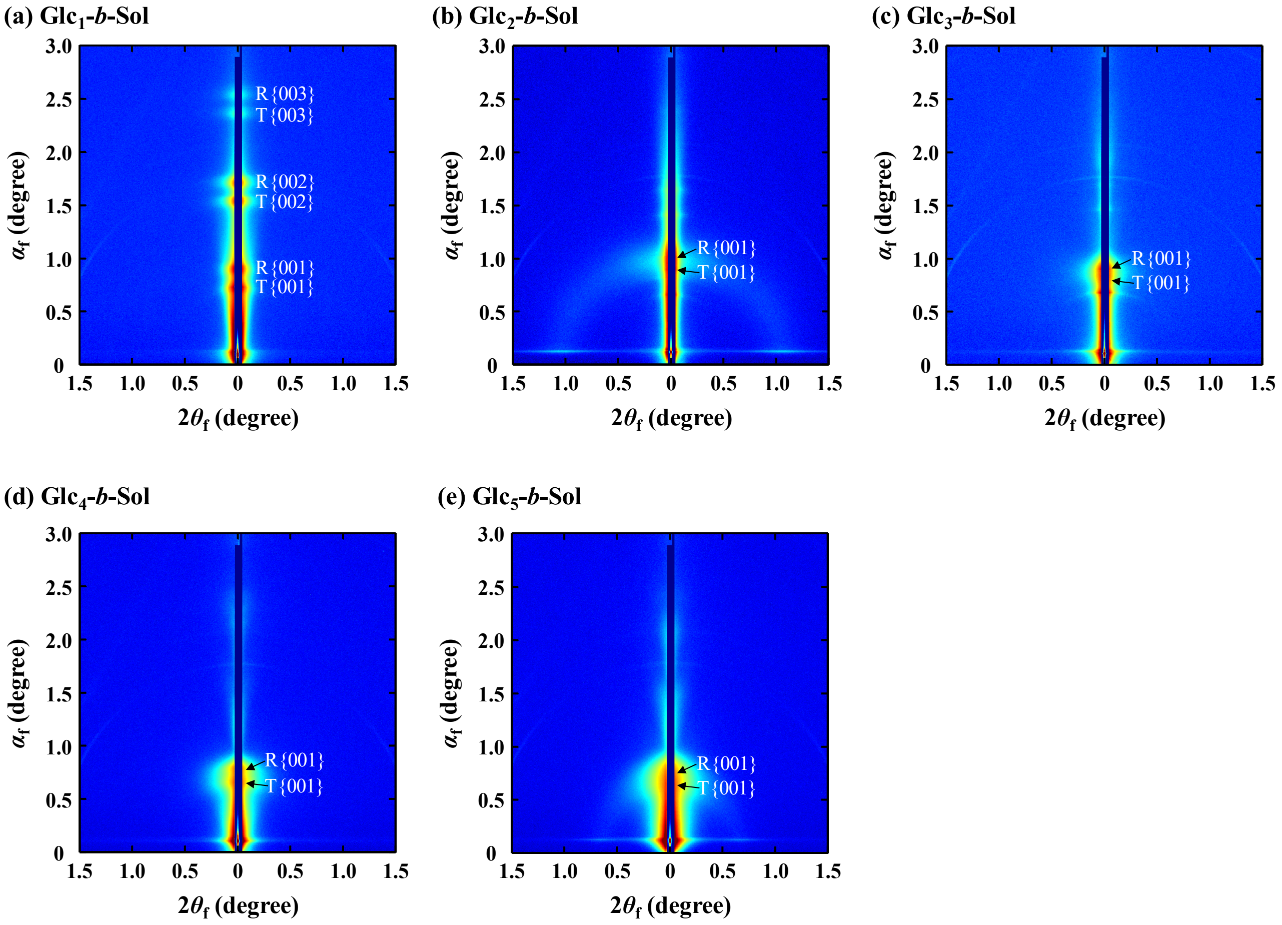

Supplementary Figure 20. GISAXS analysis of Glc$_n$-b-Sol. GISAXS data of Glc$_n$-b-Sol (n = 1 – 5) thin films after thermal annealing for 1 h (130 °C for Glc$_1$-b-Sol, Glc$_4$-b-Sol, and Glc$_5$-b-Sol; 85 °C for Glc$_2$-b-Sol and Glc$_3$-b-Sol).

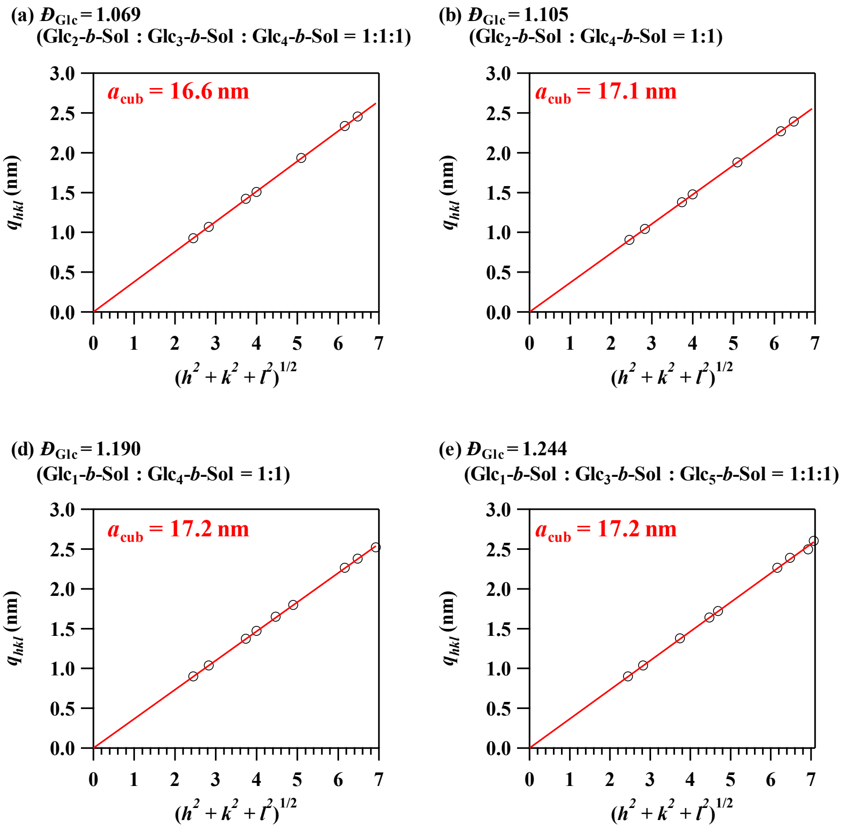

Supplementary Figure 21. SAXS analysis for GYR-forming polydisperse Glc_3-*b*-Sol samples. q_{hkl} versus $(h^2 + k^2 + l^2)^{1/2}$ plot based on the SAXS pattern observed in the GYR-forming polydisperse Glc_3-*b*-Sol samples with the DGlc of (a) 1.069, (b) 1.105, (c) 1.118, (d) 1.190, and (e) 1.244.

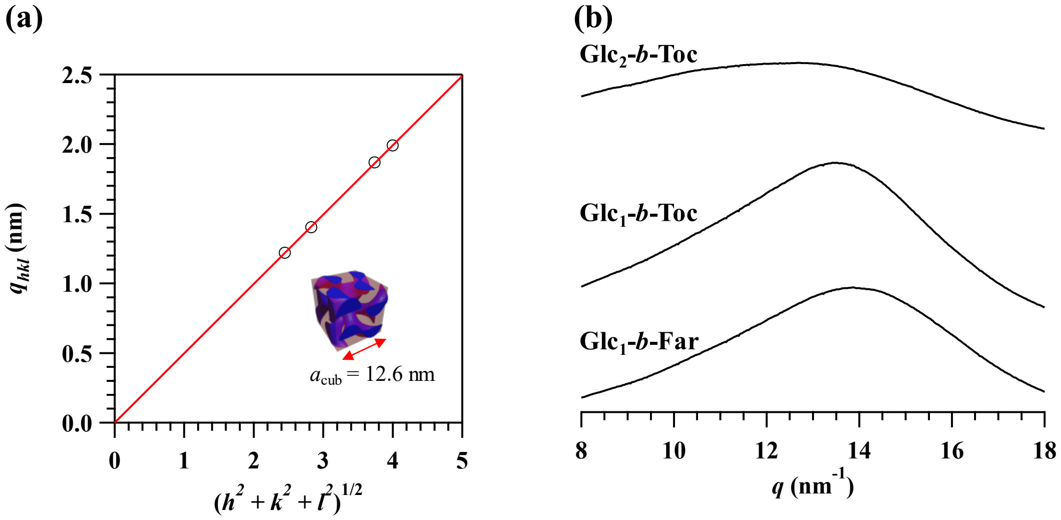

Supplementary Figure 22. X-ray analysis of tocopherol- and farnesol-Based BCOs. (a) A q_{hkl} versus $(h^2 + k^2 + l^2)^{1/2}$ plot based on the SAXS pattern observed in Glc_1-*b*-Toc. (b) WAXS profiles of (a) Glc_1-*b*-Far, (b) Glc_1-*b*-Toc, and (c) Glc_2-*b*-Toc after thermal annealing at 80 °C for 6 h.

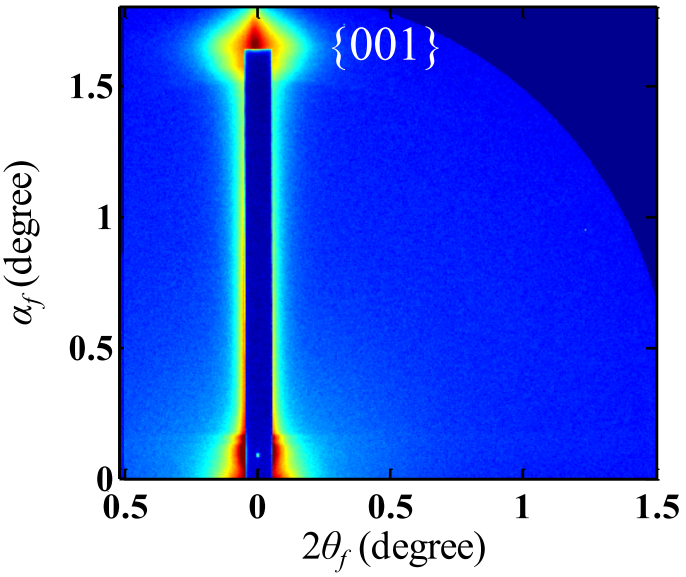

Supplementary Figure 23. GISAXS profiles of as-cast Glc$_1$-*b*-Far thin film.

Supplementary References

1. Grandjean, C.; Boutonnier, A.; Guerreiro, C.; Fournier, J.-M.; Mulard, L. A. On the Preparation of Carbohydrate – Protein Conjugates Using the Traceless Staudinger Ligation. *J. Org. Chem.*2005, *70*, 7123−7132.

2. Daly, R.; Vaz, G.; Davies, A. M.; Senge, M. O.; Scanlan, E. M. Synthesis and Biological Evaluation of a Library of Glycoporphyrin Compounds. *Chem. Eur. J.* **2012**, *18*, 14671−14670.

3. Post, E. A. J.; Fletcher, S. P. Controlling the Kinetics of Self-Reproducing Micelles by Catalyst Compartmentalization in a Biphasic System. *J. Org. Chem.* **2019**, *5*, 2741−2755.

4. Yoshida, K.; Tanaka, S.; Yamamoto, T.; Tajima, K.; Borsali, R.; Isono, T.; Satoh, T. Chain-End Functionalization with a Saccharide for 10 nm Microphase Separation: "Classical" PS-*b*-PMMA

versus PS-b-PMMA-Saccharide. *Macromolecules* **2018**, *51*, 8870−8877.

5. Otsuka, I.; Fuchise, K.; Halila, S.; Fort, S.; Aissou, K.; Pignot-Paintrand, I.; Chen, Y.; Narumi, A.; Kakuchi, T.; Borsali, R. Thermoresponsive Vesicular Morphologies Obtained by Self-Assemblies of Hybrid Oligosaccharide-*block*-poly(*N*-isopropylacrylamide) Copolymer Systems. *Langmuir* **2010**, *26*, 2325−2332.
